# Catheter ablation of atrial fibrillation in women with heart failure with preserved ejection fraction

**DOI:** 10.3389/fcvm.2024.1463815

**Published:** 2024-11-11

**Authors:** Maura M. Zylla, Johannes Leiner, Ann-Kathrin Rahm, Tobias Hoffmann, Patrick Lugenbiel, Patrick Schweizer, Christine Mages, Derliz Mereles, Meinhard Kieser, Eberhard Scholz, Hugo A. Katus, Norbert Frey, Dierk Thomas

**Affiliations:** ^1^ Department of Cardiology, Medical University Hospital, Heidelberg, Germany; ^2^ Heidelberg Center for Heart Rhythm Disorders (HCR), Medical University Hospital Heidelberg, Heidelberg, Germany; ^3^ German Center for Cardiovascular Research (DZHK), Partner Site Heidelberg/Mannheim, Heidelberg, Germany; ^4^ Institute of Medical Biometry, Heidelberg University Hospital, Heidelberg, Germany

**Keywords:** atrial fibrillation, catheter ablation, pulmonary vein isolation, women, HFpEF

## Abstract

**Background:**

Heart failure with preserved ejection fraction (HFpEF) and atrial fibrillation (AF) often coincide. Female sex is associated with both increased prevalence of HFpEF and reduced therapeutic efficacy of catheter ablation of AF. This sub-analysis of the previously published AFFECT-study evaluates outcome after cryoballoon-ablation in women with and without HFpEF.

**Methods:**

One-hundred-and-two patients (LVEF ≥ 50%) scheduled for cryoballoon-ablation of AF were prospectively enrolled. Forty-two were female. Comprehensive baseline assessment included echocardiography, stress echocardiography, six-minute-walk-test, biomarker- and quality-of-life-assessment (QoL, SF-36), and was repeated at follow-up ≥12 months after AF-ablation. Baseline parameters, procedural characteristics and outcome after AF-ablation were compared between women with and without HFpEF.

**Results:**

Women with HFpEF (*n* = 20) were characterized by higher median left atrial volume index (35.8 ml/m^2^ vs. 25.8 ml/m^2^, *P* < 0.001), left ventricular hypertrophy (median left ventricular mass index: 92.0 g/m^2^ vs. 83.0 g/m^2^, *P* = 0.027), reduced distance in the 6-min-walk-test (median: 453 m vs. 527 m, *P* = 0.008) and higher left atrial pressures (median: 14.0 mmHg vs.9.5 mmHg, *P* = 0.008) compared to women without HFpEF (*n* = 21). During follow-up, HFpEF-patients more often experienced AF-related re-hospitalization (36.8% vs. 9.1%, *P* = 0.039) and numerically higher AF-recurrence-rates (57.9% vs. 31.1%, *P* = 0.109). There was no significant improvement of heart failure-related symptoms, echocardiographic parameters and cardiac biomarkers levels. QoL showed no significant improvement in both subgroups. Women with HFpEF still exhibited a lower SF-36 Physical Component Summary Score vs. women without HFpEF (median: 41.2 vs. 52.1, *P* < 0.001).

**Conclusion:**

Women with HFpEF constitute a distinct subgroup with high rates of AF-related events after AF-ablation, and persistence of both symptoms and functional hallmarks of HFpEF. Consideration of sex-specific cardiac co-morbidities is crucial for personalization and optimization of AF-therapy.

**Clinical Trial Registration:**

ClinicalTrials.gov Identifier NCT05603611.

## Introduction

Catheter ablation of atrial fibrillation (AF) has become a widely used therapy for rhythm control with high procedural safety and acute efficacy at experienced centers (1). However, long-term therapeutic success is influenced by co-morbidities and demographic patient characteristics (2). In particular, female sex has been associated with higher rates of arrhythmia recurrence and post-procedural complications (3–6). The underlying pathomechanism of adverse outcome in women is unclear. It may be attributed to more progressed adverse atrial remodelling, differences in anatomical or substrate-related characteristics or, potentially, different referral practices (4). Women are referred for interventional therapies less often and at later time-points and older age compared to men, and rate control is chosen over rhythm control more often in female patients (4, 6). Women are also more prone to developing heart failure with preserved ejection fraction (HFpEF) (7, 8). HFpEF and AF are common co-morbidities due to shared and mutually reinforcing pathophysiological mechanisms (9, 10). Whereas the benefit of AF ablation has been shown for heart failure with reduced ejection fraction (HFrEF), the evidence regarding AF-ablation in HFpEF is heterogenous (11–14). The recently published “Catheter Ablation for Atrial Fibrillation in Preserved Ejection Fraction” (AFFECT)—study was a prospective observational study investigating the outcome in a comprehensively characterized cohort of patients with HFpEF in comparison to patients with preserved ejection fraction without heart failure (HF) (12). We were able to show that HFpEF was independently associated with reduced long-term efficacy regarding rhythm-associated endpoints and no statistically significant improvement of HF-symptoms or quality of life after cryoballoon-ablation.

Due to the adverse outcome after AF-ablation associated with female sex, we performed a subgroup analysis of the AFFECT-study regarding women with HFpEF compared to women without HF, in order to assess the impact of this important co-morbidity in women. Understanding the role of patient-specific criteria for both rhythm-associated and functional outcomes after AF-ablation is crucial for optimized therapy stratification and improving overall outcome in AF-patients.

## Material and methods

The detailed methods regarding the study have been published previously (12). In short, 587 consecutive patients scheduled for catheter ablation of AF at the Heidelberg Center of Heart Rhythm Disorders were screened from 01/2016 to 01/2019 for participation in this study. Inclusion criteria were age ≥18 years, at least one ECG-documented episode of AF and a left ventricular ejection fraction (LVEF) of ≥50%. Presence of relevant cardiac and non-cardiac co-morbidities potentially mimicking HF-symptoms constituted exclusion criteria (see Supplementary Methods Figure S1). One-hundred-and-eight eligible patients underwent baseline testing consisting of ECG, echocardiography, stress echocardiography, six-min-walk-test (6-min-WT), quality of life (QoL) assessment using the Short-Form-36 (SF-36)-questionnaire and peripheral blood tests for biomarker analyses (protocols of diagnostic tests are outlined in the Supplemental Methods section). In six patients baseline assessment revealed presence of exclusion criteria for this study. The final study cohort consisted of 102 patients of whom 42 were female (41.2%, see Supplementary Figure S1).

### Diagnosis of HFpEF

Diagnosis of HFpEF was based on the following criteria based on the current European Society of Cardiology (ESC) HF-guidelines and consensus recommendation of the Heart Failure Association (HFA) and ESC (15–17): clinical signs or symptoms of HF (NYHA ≥ II), left ventricular end-diastolic volume index (LVEDVI) ≤ 97 ml/m^2^, NTproBNP of ≥250 pg/ml in sinus rhythm or ≥600 pg/ml in AF, at least one echocardiographic sign suggestive of HFpEF (left atrial volume index (LAVI) ≥34 ml/m^2^, E/e’≥8, systolic pulmonary artery (PA) pressure via tricuspid regurgitation ≥35 mmHg, left ventricular mass index ≥95 g/m^2^ in females. Patients who had symptoms NYHA II/III but did not meet the other echocardiographic or biomarker-based criteria necessary for the diagnosis of HFpEF were stratified into the control group (Supplementary Figure S1). Importantly, diligent differentiation of HF-symptoms and predominantly AF-related symptoms in patients with persistent AF was pursued in order to confirm the diagnosis of HFpEF, and symptom re-evaluation was conducted before discharge when sinus rhythm had been restored during the ablation procedure. Furthermore, HFpEF-diagnosis was validated by the HFA-PEFF-score according to the consensus recommendation of the HFA/ESC. (16) All patients included in the HFpEF-subgroup had an HFA-PEFF-Score of ≥5 points. Patients who did not meet HFpEF-criteria represent the control group without HFpEF.

### Ablation procedure and follow-up

Second generation cryoballoon was used in all procedures (Arctic Front Advance, 28 mm, Medtronic, Minneapolis, MN) and ablation procedures were performed according to the study center's routine standards in accordance with current guidelines (see Supplementary Methods). In-house follow-up visits were scheduled at short-term (∼3 months), medium-term (∼6 months) and long-term (≥12 months) intervals. The first 3 months after the index procedure were classified as “blanking period” and arrhythmia recurrence during that time was treated with acute rhythm control by cardioversion or temporary use of antiarrhythmic drugs (AAD).

Follow-up visits included a structured interview regarding symptoms and clinical course, ECG, 24 h-holter-ECG and echocardiography. Biomarker analyses and 6-min-WT were repeated at the short-term and long-term follow-up. Stress echocardiography was repeated at the medium-term follow-up. Re-assessment of QoL was performed at the long-term follow-up. AF-recurrence was defined as any ECG-documented AF-episode >30 s. Any written reports of unscheduled medical contact during follow-up, e.g., emergency room (ER) visits and cardiac diagnostic procedures at other centers, were collected and used for confirmation of study endpoints.

The study was performed in accordance to the principles of the Declaration of Helsinki. It has been approved by local ethics committee (registration number: S-520/2015) and registered on ClinicalTrials.gov (Identifier Number: NCT04317911). Written informed consent was obtained from every patient prior to participation in this study.

### Statistical analysis

For descriptive analyses, continuous variables are reported as median with inter-quartile range (P_25_, P_75_). The Mann-Whitney-*U*-test was applied for between-group comparisons of continuous parameters and the Wilcoxon matched-pairs signed-rank-test for paired analyses within subgroups. Dichotomous variables are presented as absolute numbers and relative frequencies and were compared applying the Fisher-Boschloo-test using the R-package “exact2 × 2”, or the exact McNemar test in case of paired analyses. For time-to-event analyses, Kaplan-Meier curves were estimated and log-rank tests were performed.

Due to the exploratory character of this analysis, the *P*-values are of descriptive nature. No adjustment for multiple testing was applied. *P*-values <0.05 were denoted as statistically significant. The statistical analysis was performed using R version 4.4.0 and SPSS-version 29.0.0.

## Results

### Baseline characterization of patient cohorts

Of 102 patients included in the study, 24 were diagnosed with HFpEF. Of these, 20 patients were women (83.3%). Of 78 patients in the control group, 22 were of female sex (28.2%). There was no statistically significant difference in age or co-morbidities between the subgroups (Table 1). The majority of patients in both groups were diagnosed with paroxysmal AF, persistent AF was present in about a quarter of patients (Table 1). In accordance with the underlying cardiac condition, women with HFpEF more often described a limitation in physical capacity as classified by NYHA-scores (Table 1). Additionally, limitation in daily activity due to AF-related symptoms according to the EHRA-classification was more pronounced in women with concomitant HFpEF (Table 1). In the 6-min-WT, distance achieved was lower in patients with HFpEF (HFpEF: 453.4 m [371.0 m; 517.8 m], no HFpEF: 527.0 m [480.0 m; 564.1 m], *P* = 0.008).

**Table 1:** Baseline characteristics.

	HFpEF (*n* = 20)	No HFpEF (*n* = 22)	*P*-value
Age, median [P_25_; P_75_]	72.0 [66.0; 76, 8]	68.5 [61.0; 73.3]	0.165*
Paroxysmal AF, *n* (%)	15 (75.0)	17 (77.3)	0.946
Persistent AF, *n* (%)	5 (25.0)	5 (22.7)	0.946
CHA_2_DS_2_-Vasc-Score, *n*, %			
0	0 (0)	0 (0)	0.619**
1	0 (0)	1 (4.5)	
2	7 (35.0)	10 (45.5)	
3	5 (25.0)	6 (27.3)	
4	5 (25.0)	4 (18.2)	
5	3 (15.0)	1 (4.5)	
Body mass index, kg/m^2^	27.2 [24.4; 30.6]	25.5 [22.2; 27.5]	0.232*
Coronary artery disease, *n* (%)	3 (15.0)	5 (22.7)	0.632
Hypertension, *n* (%)	17 (85.0)	16 (72.7)	0.404
Diabetes, *n* (%)	4 (20.0)	4 (18.2)	0.998
OSAS, *n* (%)	3 (15.0)	1 (4.5)	0.382
EHRA stage			
1	0 (0)	0 (0)	**0**.026**
2	1 (5.0)	8 (36.4)	
3	15 (75.0)	13 (59.1)	
4	4 (20.0)	1 (4.5)	
NYHA stage			
I	0 (0)	14 (63.6)	**<0**.001**
II	15 (75.0)	7 (31.8)	
III	5 (25.0)	1 (4.5)	
Angina pectoris, *n* (%)	13 (65.0)	9 (40.9)	0.134
Peripheral edema, *n* (%)	15 (75.0)	9 (40.9)	**0**.**029**
AAD at baseline	5 (25.0)	7 (31.8)	0.698
Betablocker, *n* (%)	18 (90.0)	19 (86.4)	0.897
Digitalis, *n* (%)	3 (15.0)	1 (4.5)	0.382
Anticoagulation, *n* (%)	20 (100.0)	21 (95.5)	0.852
NOAC	16 (80.0)	20 (90.9)	0.423
VKA	4 (20.0)	1 (4.5)	0.175

Bold values signify those reaching statistical significance.

Fisher-Boschloo-test was used for comparisons between groups, if not stated otherwise.

* Mann-Whitney-*U*-test.

** Chi-square-test.

Rates of previous AF-related hospitalization (HFpEF: 72.7%; no HFpEF: 65.0%; *P* = 0.741) or AAD-therapy at recruitment showed no statistically significant difference between the subgroups (Table 1). Prescription of digitalis for rate control was numerically more common in women with HFpEF, however, without reaching statistical significance. The majority of patients in both groups received NOACs for oral anticoagulation therapy (Table 1).

With respect to cardiac biomarkers, NTproBNP was significantly higher in women with HFpEF (HFpEF: 605.0 ng/L [373.3 ng/L;1,568.5 ng/L]; no HFpEF: 279.5 ng/L [160.8 ng/L; 455.3 ng/L], *P* < 0.001), as well as troponin T (HFpEF: 8.0 pg/ml [5.3 pg/ml;11.8 pg/ml]; no HFpEF: 6.0 pg/ml [4.8 pg/ml; 8.3 pg/ml], *P* = 0.49). Additionally, women with HFpEF were characterized by a lower glomerular filtration rate HFpEF: 73.7 ml/min [63.3 ml/min; 82.8 ml/min] 89.2 ml/min [73.9 ml/min; 95.0 ml/min], *P* = 0.007), as well as higher levels of cystatin C (HFpEF: 1.02 mg/L [0.91 mg/L; 1.21 mg/L]; no HFpEF: 0.92 mg/L [0.79 mg/L; 1.0 mg/L] *P* = 0.026).

In baseline echocardiography, women with HFpEF displayed more progressive LA-dilation whereas ventricular dimensions showed no statistically significant difference compared to women without HFpEF (Table 2). Although preserved, left ventricular ejection fraction (LVEF) was lower in HFpEF compared to the control group without HF (Table 2). Ventricular hypertrophy was more pronounced in women with HFpEF. Whereas difference in diastolic function as quantified by E/e’ was not statistically significant at rest, women with HFpEF displayed increased E/e’ compared to women without HFpEF during stress echocardiography at maximum workload (Table 2). Additionally, longitudinal function of both left and right ventricle was diminished in women with HFpEF compared to women without HF, albeit within physiological range (Table 2). Systolic pulmonary artery pressure showed no statistically significant difference between subgroups.

**Table 2:** Echocardiographic baseline assessment.

	HFpEF (*n* = 20)	No HFpEF (*n* = 22)	*P*-value*
LA diameter, mm	43.0 [39.5; 45.8]	38.5 [36.0; 41.0]	**<0**.**001**
LA area, cm^2^	20.9 [19.1; 23.8]	16.7 [15.2; 18.8	**<0**.**001**
LAVI, ml/m^2^	35.8 [32.2; 41.9]	25.8 [22.2; 31.4]	**0**.**001**
LVEF, %	57.1 [51.3; 59.5]	59.9 [57.2; 67.5]	**0**.**003**
LV Strain, %	18.7 [16.7; 21.0], *n* = 17	20.0 [18.3; 21.1], *n* = 21	0.223
Septum, mm	12.0 [11.0; 12.8]	10.5 [10.0; 12.0]	**0**.**005**
LV mass, g	168.5 [138.3; 200.0]	148.0 [125.3; 180.5]	**0**.**017**
LV mass index, g/m^2^	92.0 [78.8; 92.0]	83.0 [70.8; 91.3]	**0**.**027**
LVEDD, mm	46.5 [42.3; 49.8]	44.0 [41.0; 48.0]	0.245
LVEDVI, ml/m^2^	36.9 [32.8; 39.6]	40.0 [31.0; 47.6]	0.158
LVESD, mm	29.5 [23.3; 32.8]	28.0 [24.8; 31.0]	0.820
sysPAP, mmHg	30.0 [25.3; 36.5], *n* = 16	29.0 [26.0; 32.0], *n* = 21	0.549
E/e’	10.0 [7.8; 13.0]	8.1 [6.3; 10.0]	0.068
MAPSE, cm	1.5 [1.0; 1.6]	1.6 [1.4; 2.0]	**0**.**045**
TAPSE, cm	2.1 [1.8; 2.4], *n* = 19	2.4 [2.1; 2.7], *n* = 21	**0**.**029**
E/e’ peak wl	9.6 [8.2; 12.6], *n* = 19	8.5 [7.1; 9.9], *n* = 20	**0**.**043**

Bold values signify those reaching statistical significance.

* Mann-Whitney-*U*-test; BP diast, diastolic blood pressure; BPsys, systolic blood pressure; LA, left atrial; LAA, left atrial appendage; LAVI, left atrial volume index; LV, left ventricular; LVEDD, left ventricular end-diastolic diameter; LVEDVI, left ventricular end-diastolic volume index; LVEF, left ventricular ejection fraction; LVESD, left ventricular end-systolic diameter; MAPSE, mitral annular plane systolic excursion; sysPAP, systolic pulmonary artery pressure; TAPSE, tricuspid annular plane systolic excursion; wl, workload. LV strain was measured as longitudinal strain with the help of the respective tool provided by GE VIVID E9.

### Procedure

Procedural parameters, in particular, procedure duration, radiation exposure, duration of cryo-energy application and local temperatures showed no statistically significant difference between women with and without HFpEF (Table 3). All identified pulmonary veins were successfully isolated in every patient. LA-pressure measured at the tip of the transseptal needle at transseptal puncture was elevated in women with HFpEF (Table 3).

**Table 3:** Procedural parameters.

	HFpEF (*n* = 20)	No HFpEF (*n* = 22)	*P*-value*
Procedure duration, min	56.5 [45.0; 83.8]	75.5 [55.0; 92.5]	0.073
Radiation duration, min	10.0 [8.0; 13.2], *n* = 19	14.4 [8.9; 21.9]	0.158
Dose area product, Gyxcm^2^	5.2 [2.4; 9.4], *n* = 19	4.6 [3.1; 10.2] (*n* = 21)	0.828
Duration of cryo-ablation, min	14.0 [12.0; 22.0], *n* = 19	15.2 [13.0; 21.0] (*n* = 76)	0.487
Average temperature, °C	−46.0 [−47.7; −43.9]	−45.0 [−47.7;−42.9]	0.472
Mean LA pressure, mmHg	14.0 [10.0; 18.8]	9.5 [6.3; 11.0] (*n* = 20)	**0**.**026**
Procedural complication^a^, *n* (%)	1.0 (5.0)	1.0 (4.5)	1.00**

Bold values signify those reaching statistical significance.

* Mann-Whitney-*U*-test.

** Fisher-Boschloo-test.

^a^ Pericardial effusion without gemodynamic compromise or need for intervention, CTI, cavotricuspid isthmus ablation.

The incidence of procedure-related complications was low. In two cases pericardial effusion was detected after the procedure, without hemodynamically compromise or need for intervention. These findings were be attributed to inflammatory reactions and pericardial effusion regressed after anti-inflammatory therapy (Table 3).

### Long-term follow-up

**Figure 1:**
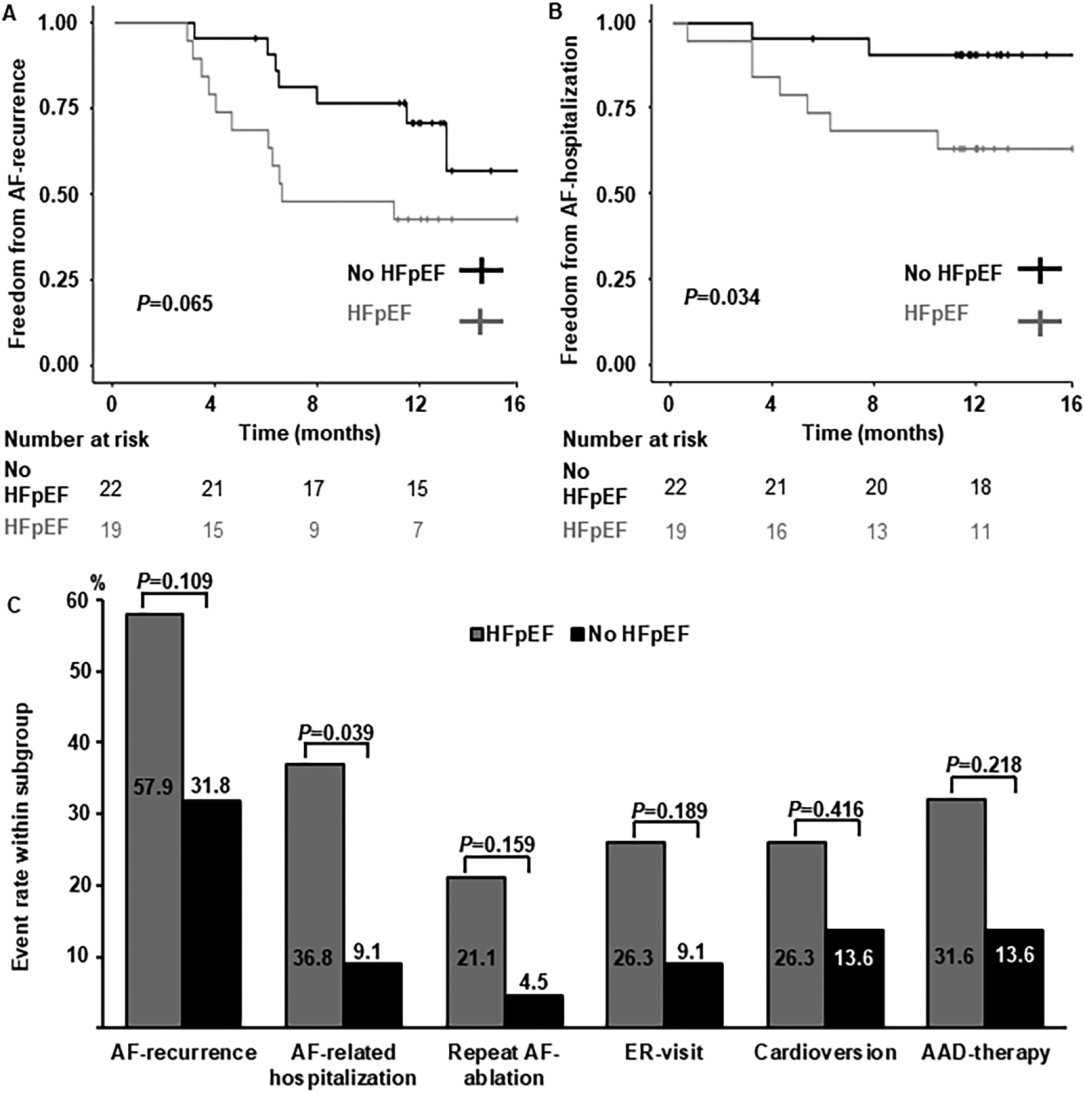
Arrhythmia-related long-term follow-up. **(A)** Kaplan-Meier curves depicting freedom from AF-recurrence in women with HFpEF and without heart failure. **(B)** Kaplan-Meier curves depicting freedom from AF-related hospitalization in women with HFpEF and without heart failure. **(C)** Comparison of arrhythmia-related endpoints. Percentages per subgroup are indicated within or above columns. AAD, antiarrhythmic drug; ER, emergency room.

Long-term follow-up rates were high in both groups (HFpEF: *n* = 19, 95.0%; no HFpEF: *n* = 22, 100%) and median follow-up duration was 386 days (HFpEF: 393 days [373 days; 472 days]; no HFpEF: 384 days [365 days; 418 days], *P* = 0.367). Rates of AF-recurrence, repeat-ablation, cardioversion, ER-visits and continuation of AAD-therapy were numerically higher in women with HFpEF, without reaching statistical significance (Figures 1A,C). There was a statistically significant difference of AF-related re-hospitalization, with a four-fold increase of re-hospitalization rates HFpEF compared to women without HF (Figures 1B,C).

**Figure 2:**
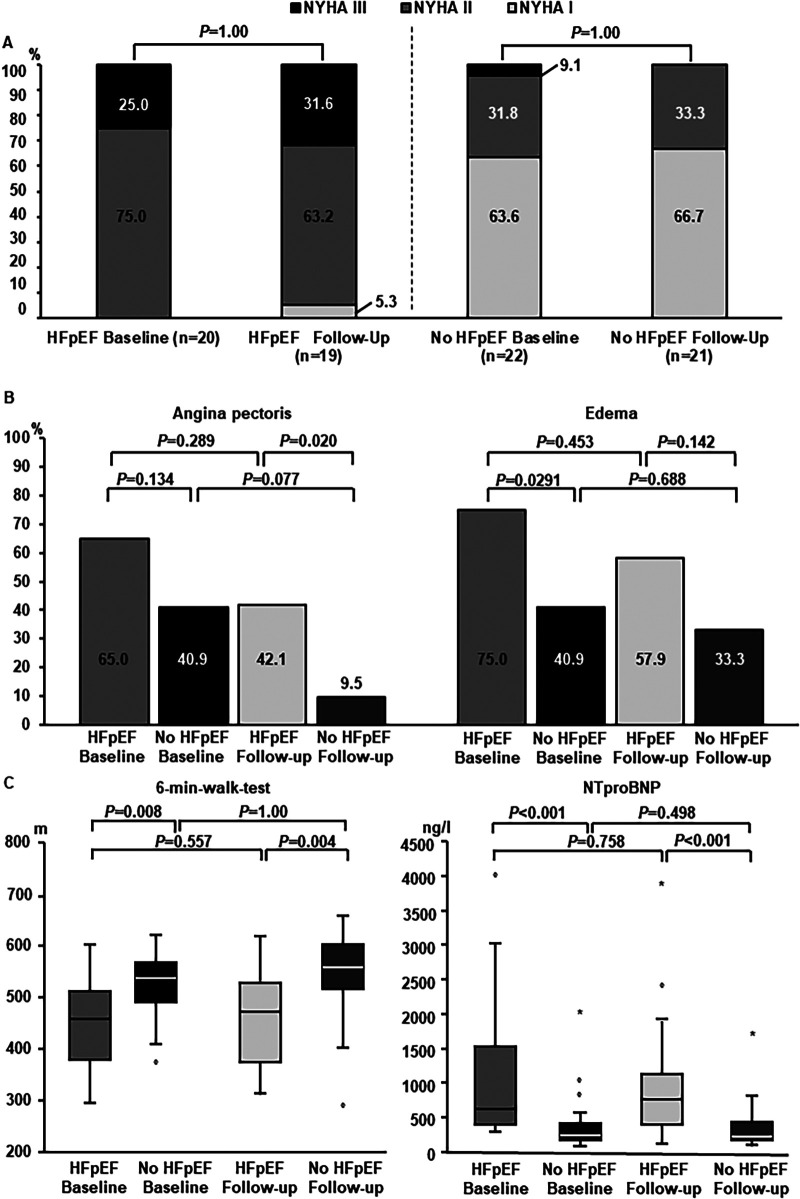
Heart failure-related long-term follow-up. **(A)** Distribution of NYHA-states at baseline and long-term follow-up. Left columns: women with HFpEF. Right columns: women without HFpEF. For statistical comparison by McNemar-test subgroups with NYHA II and NYHA III were summarized. **(B)** Cardiac symptoms at follow-up. Subgroup comparisons were performed between women with and without HFpEF, as well as between baseline and long-term follow-up within subgroups. **(C)** Change in heart-failure related biomarkers and 6-min-walktest. Left panel: Performance 6-min-walktest at baseline and follow-up in women with and without HFpEF. Right panel: NTproBNP-levels at baseline and follow-up in women with and without HFpEF.

With respect to NYHA-class, no statistically significant change after ablation could be detected in both groups (Figure 2A). There was a reduction in angina pectoris in both groups which was more pronounced in women without HFpEF and not statistically significant in women with HFpEF. At follow-up, women with HFpEF more often described persistent angina pectoris compared to women without HF (Figure 2B). Reduction in peripheral edema was not statistically significant in both subgroups after AF-ablation compared to baseline assessment (Figure 2B). Furthermore, there was no statistically significant improvement in walking distance achieved in the 6-min-WT in women with or without HFpEF and differences between these two subgroups persisted (Figure 2C). Similarly, NTproBNP-levels remained elevated in women with HFpEF compared to the control group, without statistically significant reduction after AF-ablation (Figure 2C).

In echocardiographic long-term follow-up, women with HFpEF showed progressive LA-enlargement in contrast to women without HFpEF (Table 4). Left ventricular systolic, diastolic and longitudinal function remained stable in both groups compared to baseline (Table 4). In both groups there was a small increase in septal diameters compared to baseline. However, the absolute changes were not within a clinically relevant range (Table 4). There was not clinically relevant change in LV-mass index and women with or without HFpEF compared to baseline, and LV-mass index was still elevated in HFpEF in comparison to women without HF (Table 4).

**Table 4:** Echocardiographic long-term follow-up.

	HFpEF baseline (*n* = 20)	HFpEF 12 Mo FU (*n* = 19)	*P*-value*	No HFpEF baseline (*n* = 22)	No HFpEF 12 Mo FU (*n* = 21)	*P*-value*	*P*-value**
LAVI, ml/m^2^	35.8 [32.2; 41.9]	40.9 [36.3; 53.9]	**0.018**	25.8 [22.2; 31.4]	29.3 [24.7; 34.5]	0.566	**<0**.**001**
LVEF,%	57.1 [51.3; 59.5]]	56.3 [51.9; 62.7]	0.879	59.9 [57.2; 67.5]	59.7 [57.0.5; 63.6]	0.357	0.269
Septum, mm	12.0 [11.0; 12.8]	12.5 [11.0; 13.0]	**0.011**	10.5 [10.0; 12.0]	11.0 [10.0; 11.0]	0.570	**<0**.**001**
LV mass index, g/m^2^	92.0 [78.8; 92.0]	92.5 [81.5; 110.3]	0.257	83.0 [70.8; 91.3]	76.0 [72.0; 81.5]	0.578	**0**.**002**
sysPAP, mmHg	30.0 [25.3; 36.5], *n* = 16	32.0 [30.3; 39.0] (*n* = 16)	0.068 (*n* = 16)	29.0 [26.0; 32.0], *n* = 21	31.0 [27.0; 36.5] (*n* = 20)	0.227	0.249
E/e’	10.0 [7.8; 13.0]	10.5 [8.4; 12.1] (*n* = 18)	0.983 (*n* = 18)	8.1 [6.3; 10.0]	7.5 [6.2; 11.3]	0.563	0.057
MAPSE, cm	1.5 [1.0; 1.6]	1.5 [1.3; 1.9] (*n* = 17)	0.077 (*n* = 17)	1.6 [1.4; 2.0]	1.7 [1.5; 2.0] (*n* = 20)	0.726	0.193

Bold values signify those reaching statistical significance.

* Wilcoxon matched-pairs signed-rank test (comparisons within subgroups).

** Mann-Whitney-*U*-test comparing patients with and without HFpEF at long-term follow-up; LAVI, left atrial volume index; LV, left ventricular; LVEF, left ventricular ejection fraction; MAPSE, mitral annular plane systolic excursion; sysPAP, systolic pulmonary artery pressure.

**Figure 3:**
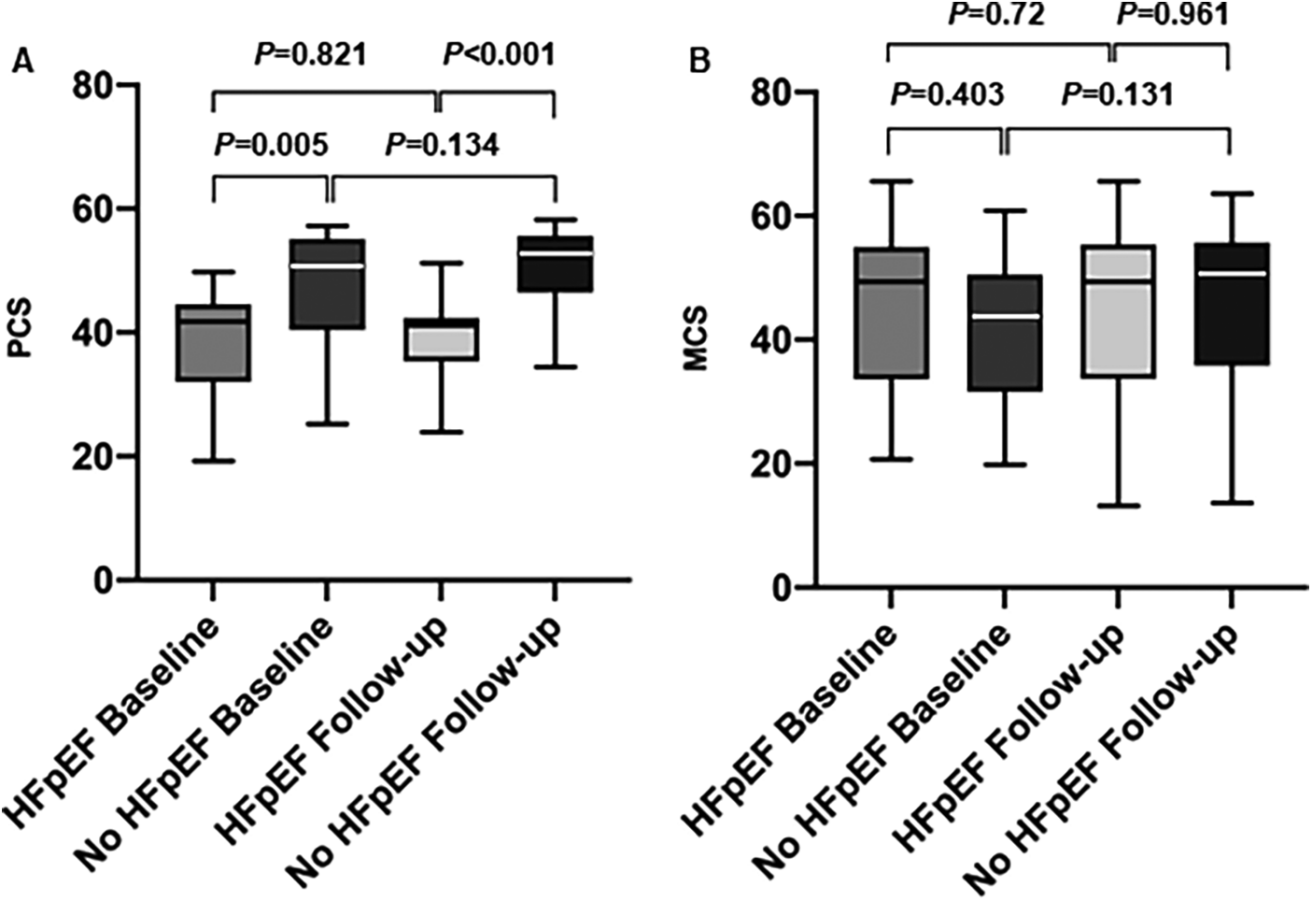
Quality of life assessment (SF-36). **(A)** Physical component summary scales (PCS) before and after AF-ablation in women with and without HFpEF. **(B)** Mental component summary scales (MCS) before and after AF-ablation in women with and without HFpEF.

With respect to QoL, women with HFpEF displayed lower scores in the SF-36 (SF-36) physical summary (PCS) scale at baseline in comparison to the control group without HFpEF (Figure 3A). There was no statistically significant difference regarding the mental component summary (MCS) scores between the two groups (Figure 3B). In both groups, no statistically significant improvement of PCS or MCS scores could be detected after initially successful PVI (Figure 3) and women with HFpEF still displayed lower PCS scores compared to women without HF.

## Discussion

Atrial fibrillation and HFpEF are two common and often coinciding conditions due to shared risk factors and mutually reinforcing pathophysiology (10). Prevalence of HFpEF is particularly elevated in women and may be crucial for individual prognosis and therapeutic outcome in other concomitant cardiac conditions (8).

In our single-center study investigating outcome after cryoballon-ablation in a comprehensively characterized HFpEF-cohort of male and female patients compared to patients without HF, we have previously shown that patients with HFpEF had a higher probability of AF-recurrence, persistent cardiac symptoms and QoL-impairment after cryoballoon-ablation (12). In contrast, patients without HFpEF showed improvement of both symptoms and QoL at long-term follow-up after AF-ablation. Statistical adjustment for intergroup differences in sex did not alter the overall results regarding rhythm-associated and clinical outcome in the AFFECT study.

Adding to this previous evidence, this sub-analysis focuses on women in the AFFECT study cohort. Co-existence of HFpEF in women with AF was particularly associated with reduced therapeutic efficacy and a higher rate of AF-related re-hospitalization. We show that presence of HFpEF in women is associated with more pronounced AF-related symptoms at baseline. Lack of symptomatic improvement at follow-up was reflected in persistent elevation of HF-related biomarkers and reduced distance in the six-min-walk-test. In contrast to the mixed-sex cohort, both women with and without HFpEF described no statistically significant improvement of quality of life after AF-ablation. Additionally, women with HFpEF showed persistently lower scores in the PCS than women without HF. AF-recurrence, repeat AF-ablation, ER-visits, repeat cardioversion and continuation of AAD was numerically more common in women with HFpEF. Whereas intergroup differences may be of clinical significance, the limited number of patients in this analysis may be a reason for failure to reach statistical significance.

There was no significant difference between the two subgroups regarding other co-morbidities potentially affecting outcome after AF-ablation. Thus, HFpEF and its associated effects on LA-remodelling and hemodynamics reflected in increased LA-pressure and LA-dilation seem to constitute the main risk factors for reduced therapeutic success in this cohort. Therefore, this sub-analysis identifies women with HFpEF as a patient subgroup at risk for pronounced symptomatic and functional limitation in the context of AF. It highlights the need for early and optimized therapy in this subgroup, as well as the relevance of sex-specific distribution and prevalence of additional cardiac co-morbidities for AF-therapy.

Importantly, AF-ablation in women without HF was associated with an alleviation of some cardiac symptoms, however, without significant improvement of QoL. This stands in contrast to our previous analysis of mixed-sex cohorts, illustrating a potential attenuation of benefits regarding clinical outcome after AF-ablation in women, irrespective of presence of HFpEF. We cannot exclude that detection of relevant improvement in QoL or other clinical parameters was limited due to the small cohort size. However, previous sex-specific analyses of outcome after AF-ablation have also pointed out reduced therapeutic success associated with female sex (3, 5). Presence of AF in women is associated with longer and more symptomatic AF-episodes, higher ventricular rates and greater impairment of quality of life (4). Additionally, women more often suffer from adverse events under medical antiarrhythmic therapy (18). Subgroup analyses of large-scale randomized trials and large registries have shown higher rates of AF-recurrence and re-hospitalization in women after catheter ablation of AF (3, 5). Furthermore, women were more likely to experience periprocedural complications, in particular vascular or bleeding complications (4). Various reasons for adverse outcome associated with the female sex have been discussed. In comparison to men, women are referred for interventional therapies less often and at later stages of the disease potentially predisposing for progressed atrial remodelling at the time of referral (4). More advanced atrial fibrosis and higher prevalence of extra-pulmonary triggers in women may contribute to less favourable clinical outcome of AF-ablation (19). In the FIRE and ICE trial, reduced efficacy in women was observed both in radiofrequency- and cryoballoon-ablation approaches (5). A recent sex-specific analysis of the MANIFEST-registry demonstrated no significant difference in outcome between male and female patients after pulsed field ablation of AF (20). However, a potential underlying mechanism attenuating sex-specific differences in modern ablation technologies in has to be confirmed and explored in future studies.

We observed longer procedure duration and fluoroscopy times in the subgroup without HFpEF, however, with high variability and without reaching statistical significance, possibly due to the limited cohort size. As the numeric differences in procedure duration may be of clinical significance, procedural aspects in this patient group be investigated in future, adequately powered trials. With respect to complications, two cases of pericardial effusion occurred in the AFFECT study cohort of 102 patients, both in women and in both cases they were caused by inflammatory reactions. In contrast to previous sex-specific analyses, there was no increased incidence of bleeding or vascular complications (3). Higher rates of pericarditis after AF-ablation in women have been described in large population-based analyses of administrative data (21). However, underlying mechanisms of this higher risk in women, particularly in the context of cryoballoon-ablation, are yet unknown.

In heart failure with reduced ejection fraction (HFrEF), beneficial effects of AF-ablation have been shown by randomized trials (11). Current evidence regarding outcome after AF-ablation in HFpEF is heterogenous. In comparison to medical therapy, AF-ablation in HFpEF improved clinical outcome and reduced heart failure hospitalization in retrospective propensity-score matched analyses (13, 22). One small randomized-controlled trial in patients with HFpEF showed improvement of hemodynamic characteristics in invasive measurements after AF-ablation compared to medical therapy (23). Large-scale randomized-controlled trials on AF-ablation in HFpEF are still being awaited. The “CAtheter-Based Ablation of atrial fibrillation compared to conventional treatment in patients with Heart Failure with Preserved Ejection Fraction”-trial (CABA-HFPEF-DZHK27) is currently enrolling and will investigate the effect of early AF-ablation on cardiovascular outcomes in HFpEF and HFmrEF. Whereas the AFFECT-study primarily focused on rhythm-associated and QoL outcome parameters in HFpEF, CABA-HFPEF-DZHK27 is designed to evaluate prognostic endpoints including cardiovascular death, stroke, and total unplanned hospitalizations due to HF or acute coronary syndrome in a large patient cohort. Compared to CABA-HFPEF-DZHK2, inclusion criteria for the HFpEF-subgroup were stricter in our monocentric study and association of symptoms with HF rather than only with AF-episodes was diligently assessed, resulting in a smaller patient cohort and insufficient statistical power to evaluate prognostic endpoints.

In our prospective study and particularly the subgroup analyses on women with HFpEF, the proposed beneficial effects of AF-ablation were not observed. Differences in outcomes between studies may be explained by different patient selection criteria, as well as different criteria qualifying for HFpEF-diagnosis. In our prospective study, we pursued thorough evaluation of HF-symptoms and confirmation of their temporal association with AF-episodes, used higher cut-offs for HF-related biomarkers and additionally validated the patient selection for the HFpEF-subgroups by current diagnostic HFpEF-scores. Presence of HFpEF-characteristics was also confirmed by invasive measurements of LA-pressure, and were evident in measurable impaired performance in the six-min-walk-test. Due to our strict selection criteria, the cohort size of HFpEF-patients in this study is small. However, the comprehensive characterization of patients employing multiple diagnostic modalities and a high rate of successful long-term follow-up constitute strengths of the study and this sub-analysis.

Subgroup analyses of the Early Treatment for Atrial Fibrillation for Stroke Prevention Trial (EAST-AFNET4) have pointed to beneficial effects of early rhythm control with respect to cardiac events, hospitalization and progression of HF-symptoms in both HFrEF- and HFpEF-patients with additional cardiovascular risk factors (24). Unfortunately, the duration of previous AF in our cohort was not documented. However, optimizing referral practices aiming for early rhythm-control in women and particularly women with additional HFpEF may assist in prohibiting additional hemodynamically compromising effects on LA-remodelling which we observed in our HFpEF-cohort. Furthermore, we did not compare AF-ablation to medical therapy in HFpEF. Thus, catheter ablation of AF in women with HFpEF may still result in favorable clinical outcome in comparison to conservative treatment and should be further evaluated, particularly in the light of new technologies for AF-ablation emerging. Even though the results of this study can only be interpreted as hypothesis-generating, it may provide a basis for future trials which should strive to select a well-defined HFpEF-cohort, particularly as current evidence is based on heterogenous selection criteria. Importantly, a recent meta-analysis comparing outcome after AF-ablation in patients according to HF-phenotype and including 12 randomized-controlled trials with 2,465 participants showed a reduced risk of HF-events in patients with HFrEF after catheter ablation of AF but limited or no benefit in HFpEF (14). This confirms the notion delivered by our analysis.

Due to potentially more pronounced LA-remodelling in HFpEF (25, 26), outcome after different ablation approaches and potentially also targeting extra-pulmonary-vein targets may differ from outcome after cryoballoon-ablation. Extra-pulmonary targets may be of relevance particularly in women (19). Outcome after AF-ablation in HFpEF employing different ablation approaches and energy sources, including pulsed field ablation, should be investigated in future prospective studies in order to identify optimized strategies tailored to sex-specific and co-morbidity-related characteristics and risk factors.

### Limitations of the study

The monocentric design and relatively small cohort size—in part resulting from strict selection criteria especially for the HFpEF-subgroup—constitute main limitations of this study. Inclusion criteria in previous studies on AF-ablation in HFpEF are highly heterogenous. This relates to different cutoff-values chosen for LVEF, NTproBNP, echocardiographic parameters as well as evaluation of HF-symptoms. In addition to comprehensive baseline evaluation with different diagnostic modalities, diligent prospective clinical evaluation in each case enabled us to clearly differentiate cardiac symptoms only present during AF-episodes from “true” HF-symptoms irrespective of presence of AF. By employing our rigorous selection criteria, we have identified a distinct subgroup of HFpEF-patients, mirrored in associated functional characteristics. However, future large-scale projects are needed to extend and validate our results which should be regarded as hypothesis-generating. Unfortunately, comparing outcome between men and women within the two subgroups was not possible due to the low number of male patients (*n* = 4) in the HFpEF-subgroup.

The mixed population of patients with both paroxysmal and persistent AF may have impacted the rhythm-associated outcome in this study. However, this study was designed to investigate a real-world cohort from a high-volume ablation center, which is reflected in the distribution of different AF-types. Unfortunately, separate analyses of paroxysmal and persistent AF in this sub-analysis were not possible due to the limited cohort size.

Arrhythmia-related follow-up in this study relied on 24 h-holter and incidental symptom-related ECG-assessment, and no implantable loop recorders or wearables were used. Therefore, evaluation of AF-burden was not feasible and asymptomatic AF-episodes may have been missed.

LA-dilation is both a morphological hallmark of HFpEF and a risk factor for AF-recurrence, limiting independent evaluation of bias due to differences in progression of LA-remodelling between subgroups.

Unfortunately, detailed information on glomerular filtration rate, administration of heart failure medication and diuretics has not been evaluated for this cohort. Additionally, sodium glucose cotransporter 2-(SGLT2)-inhibitors had not yet been established as therapy in HFpEF at the time of recruitment. Therefore, potential beneficial effects of this therapy in patients with HFpEF and AF could not be investigated and may play a role in contemporary HFpEF-cohorts.

## Conclusion

In this real-world cohort, women with HFpEF more often exhibited AF-related re-hospitalization after cryoballoon-ablation of AF. HF-related symptoms, cardiac biomarkers, and reduced performance in functional tests showed no improvement after AF-ablation. Both women with and without HFpEF did not describe improvement of QoL at follow-up. Both sex-specific and co-morbidity-related pathophysiological characteristics may affect outcome after AF-ablation in women and should be evaluated in future investigations for development of tailored and optimized therapeutic strategies.

## Data Availability

The raw data supporting the conclusions of this article will be made available by the authors, without undue reservation in accordance with applicable personal data protection regulations.
