# Supplementary material for: Catheter ablation of atrial fibrillation in women with heart failure with preserved ejection fraction
Source: Front Cardiovasc Med. 2024 Nov 11;11:1463815. doi: 10.3389/fcvm.2024.1463815 (PMC11586357; doi:10.3389/fcvm.2024.1463815)
Supplement: Supplementary file 1 [file Datasheet1.docx]

**Catheter ablation of atrial fibrillation in women with heart failure with preserved ejection fraction**

**SUPPLEMENTAL METHODS**

**Patient selection**

Exclusion criteria for participation in this study were cardiac and non-cardiac conditions mimicking heart failure symptoms and other criteria precluding catheter ablation or study-associated diagnostic tests:

• Aortic or mitral stenosis >I° or regurgitation >II°

• Coronary artery disease with stenoses of >70% or need for PCI at the time of recruitment

• PCI within 60 days prior to recruitment

• Heart surgery (CABG, valve replacement or repair) within 90 days prior to recruitment

• Presence or previous diagnosis of dilated, hypertrophic or restrictive cardiomyopathy

• Previously diagnosis of reduced left ventricular ejection fraction in sinus rhythm

• Presence of cardiac storage disease (e.g. amyloidosis, Fabry’s disease)

• Presence of hemodynamically relevant congenital heart defect

• Pericardial disease

• Diagnosis of primary pulmonary hypertension

• Severe chronic obstructive pulmonary disease with need for home oxygen therapy, oral steroid therapy, hospitalization due to exacerbation within 12 months prior to recruitment or severe pulmonary disease based to clinical evaluation

• Other non-cardiac conditions associated with limited physical capacity (morbid obesity, severe anemia)

• Pregnancy

• Cardiac assist device or heart transplantation in history

• Other limitations for adequate performance of stress echocardiography (e.g. orthopedic reasons leading to impaired mobility)

**Baseline evaluation**

The six-minute walk test was performed according to guidelines.^S1^

Peripheral blood samples were collected after a period of rest of at least 20 minutes to account for susceptibility of NTproBNP-values to physical activity. Clinical routine measurements were performed by the central laboratory of Heidelberg University Hospital.

In case of signs of previously undiagnosed cardiomyopathy or myocardial ischemia in echocardiography or stress echocardiography, further invasive or non-invasive tests were performed according to the respective clinical guidelines by the European Society of Cardiology (ESC).

Of the initial cohort of 108 patients, six patients were excluded from the study based on findings during baseline assessment (previously undiagnosed hypertrophic cardiomyopathy in one patient, relevant mitral regurgitation in one patient, one patient showed signs of myocardial ischemia in stress echocardiography and underwent PCI, two patients received radiofrequency ablation instead of cryoballoon ablation and in one patient the ablation procedure was not feasible because of vascular access problems due to bilateral iliac vein thromboses).

**Echocardiography and stress echocardiography**

Echocardiography and stress echocardiography were performed according to current guidelines of the American Society of Echocardiography (ASE) using GE VIVID E9 (GE Healthcare, Chicago/Illinois, USA) and Philips Affiniti 30 (Koninklijke Philips N.V., Amsterdam, Netherlands). All examinations were carried out by physicians proficient in echocardiography and interpretation of respective measurements. Results were additionally re-assessed by a second expert who was blinded for the respective subgroup stratification. In patients who had been in AF during baseline echocardiographic evaluation, repeat measurements of diastolic function and longitudinal strain were performed in sinus rhythm before discharge after the index procedure to enhance diagnostic accuracy.

The protocol applied for stress echocardiography was based on current guidelines and previous investigations on exercise testing in patients with HFpEF to allow for evaluation of diastolic function both in submaximal and maximal exertion during stress testing.^S2,S3^ Under continuous ECG-monitoring, patients cycled at 60rpm starting at a workload of 25W. Blood pressure and heart rate were documented at two minutes-intervals. Workload was increased every two minutes by 10-W-increments until a heart rate of 90 to 100/min was reached (“submaximum workload”). If symptoms, LV-function echocardiography and ECG allowed for continuation of stress testing, the workload was further increased in 25-W-increments every two minutes until the individually possible maximum workload was reached for further two minutes. Echocardiographic assessment of LV-contractility, diastolic function and systolic PA-pressure was performed at rest before stress testing, at submaximum workload and maximum workload as well as post-exercise as soon as the heart rate normalized to below 100/min. With respect to all echocardiographic characteristics, measurements were excluded when quality of individual patient-specific echocardiographic conditions did not allow for unequivocal assessment.

**Quality of Life (QoL) assessment**

The SF-36-questionnaire was used for measuring QoL before and at long-term follow-up after PVI. The effect of cryoballoon-ablation of AF on QoL was assessed in patients who completed the SF-36 questionnaire both at recruitment and at long-term follow-up. Eight main subscales - physical functioning (pfi), physical role functioning (rolph), pain (pain), general health perception (ghp), vitality (vital), social role functioning (social), emotional role function (rolem), and mental health (mhi) - were recorded. Two component summary scores were calculated: physical component summary score (PCS) and mental component summary score (MCS), ranging from 0 (worst possible health) to 100 (best possible health). Analyses of QoL-assessment were performed in accordance to instructions by the German version of the SF-36 questionnaire and using provided SPSS-syntaxes for calculation of subscales and summary scores.^S4^ The factor coefficients of a German normative sample from 1994 were used for calculating PCS and MCS as proposed by the publishers of the German SF-36-questionnaire.^S4^

**Ablation procedure and periprocedural management**

Prior to PVI, patients underwent transesophageal echocardiography (TOE) to rule out LA-thrombus. Patients on NOACs were advised to pause anticoagulation medication 24h prior to the procedure. In case of therapy with vitamin K antagonists (VKA) therapy was continued with a target INR of 2.0 – 2.5 at the time of procedure. Second generation cryoballoon was used in all procedures (Arctic Front Advance, 28mm, Medtronic, Minneapolis, MN). The procedure was conducted under conscious sedation. The right femoral vein was used as access site. A quadripolar diagnostic catheter was placed in the coronary sinus (6F ELA Xtreme, 6F). After single transseptal puncture, the cryoballoon was advanced to the LA via a steerable sheath (12F Flexcath Advance, Medtronic, Minneapolis, MN). A spiral mapping catheter (20 mm Achieve Advance, Medtronic, Minneapolis, MN) was advanced through the inner lumen of the cryoballoon and placed at the pulmonary vein (PV) ostia. After inflation of the cryoballoon, PV-angiography was performed for assessment and optimization of occlusion before cryoablation for a target time of 180 seconds per vein. PV potentials were recorded during ablation with the help of the mapping catheter. Prolongation of ablation times or multiple freezes were performed if necessary when the first freeze die not results in sufficient PVI. Temperature of the surrounding tissues was monitored by an esophageal probe. During ablation of the right pulmonary veins, phrenic pacing was performed for monitoring of phrenic nerve function. Pericardial effusion was excluded by echocardiography immediately after the procedure and before discharge. Oral anticoagulation with NOACs was resumed one day after the procedure.

**Follow-Up**

Follow-up was performed at short-term (~3 months), medium-term (~6 months) and long-term (≥12 months) intervals. In some patinets, long-term follow-up visits had to be re-scheduled due to individual personal reasons. Due to the overall limited sample size, we included these slightly “delayed” long-term follow-up timepoints in the analyses and comparisons between groups.

**SUPPLEMENTAL REFERENCES:**

S1. ATS Committee on Proficiency Standards for Clinical Pulmonary Function Laboratories. ATS statement: guidelines for the six-minute walk test. *Am J Respir Crit Care Med*. 2002; **166**:111-7.

S2. Lancellotti P, Pellikka PA, Budts W, Chaudhry FA, Donal E, Dulgheru R, Edvardsen T, Garbi M, Ha J-W, Kane GC, Kreeger J, Mertens L, Pibarot P, Picano E, Ryan T, Tsutsui JM, Varga A. The clinical use of stress echocardiography in non-ischaemic heart disease: recommendations from the European Association of Cardiovascular Imaging and the American Society of Echocardiography. *Eur Heart J Cardiovasc Imaging*. 2016; **17**:1191-1229.

S3. Borlaug BA, Nishimura RA, Sorajja P, Lam CSP, Redfield MM. Exercise Hemodynamics Enhance Diagnosis of Early Heart Failure With Preserved Ejection Fraction. Circ Heart Fail. 2010; **3**:588-595.

S4. Morfeld M, Kirchberger I, Bullinger M. SF-36, Fragebogen zum Gesundheitszustand. 2. ergänzte und überarbeitete Auflage. *Hogrefe Verlag*. 2011.

S5. Austin PC. An introduction to propensity score methods for reducing the effects of confounding in observational studies. *Multivar Behav Res.* 2011;**46**:399–424.

**SUPPLEMENTAL FIGURES**

**Figure S1**

**
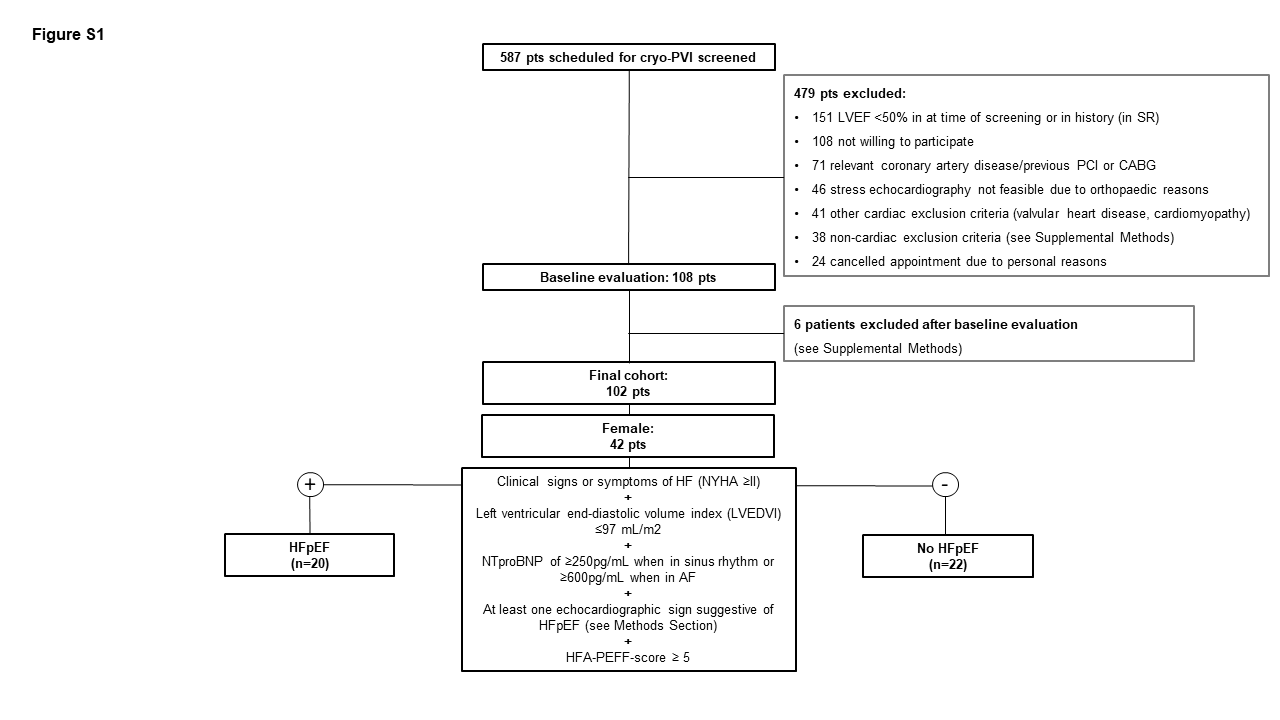
**

**SUPPLEMENTAL FIGURE LEGEND**

**Figure S1: Enrolment flowchart.** Screening and recruitment, as well as reasons for exclusion from the study are shown. Diagnostic criteria for HFpEF and respective subgroup stratification are depicted. Pts=patients
